# Effectiveness of clinical scores in predicting coronary artery disease in familial hypercholesterolemia: a coronary computed tomography angiography study

**DOI:** 10.1007/s11547-023-01610-z

**Published:** 2023-03-06

**Authors:** Federica Catapano, Nicola Galea, Giacomo Pambianchi, Laura D’Erasmo, Cristian Borrazzo, Giulia Cundari, Livia Marchitelli, Marianna Maranghi, Ilenia Minicocci, Alessia Di Costanzo, Iacopo Carbone, Marco Francone, Marcello Arca, Carlo Catalano

**Affiliations:** 1 Present Address: Department of Radiological, Oncological and Pathological Sciences, Sapienza University of Rome, Viale Regina Elena 324, 00161 Rome, Italy; 2 Department of Biomedical Sciences, Humanitas University, Via Rita Levi Montalcini 4, 20072 Pieve Emanuele, Milan, Italy; 3 IRCCS Humanitas Research Hospital, Via Manzoni 56, 20089 Rozzano, Milan, Italy; 4 Department of Experimental Medicine, Sapienza University of Rome, Viale Regina Elena 324, 00161 Rome, Italy; 5 Translational and Precision Medicine, Sapienza University of Rome, Viale Regina Elena 324, 00161 Rome, Italy; 6 Department of Medical-Surgical Sciences and Biotechnologies, Sapienza University of Rome, Corso Della Repubblica 79, 04100 Latina, Italy

**Keywords:** Familial hypercholesterolemia, Atherosclerotic cardiovascular disease, Coronary computed tomography angiography, CAD-RADS, Dutch lipid clinic network score, FH risk score, SAFEHEART-RE

## Abstract

**Purpose:**

One of the major challenges in the management of familial hypercholesterolemia (FH) is the stratification of cardiovascular risk in asymptomatic subjects. Our purpose is to investigate the performance of clinical scoring systems, Montreal-FH-score (MFHS), SAFEHEART risk (SAFEHEART-RE) and FH risk score (FHRS) equations and Dutch Lipid Clinic Network (DLCN) diagnostic score, in predicting extent and severity of CAD at coronary computed tomography angiography (CCTA) in asymptomatic FH.

**Material and methods:**

One-hundred and thirty-nine asymptomatic FH subjects were prospectively enrolled to perform CCTA. MFHS, FHRS, SAFEHEART-RE and DLCN were assessed for each patient. Atherosclerotic burden scores at CCTA (Agatston score [AS], segment stenosis score [SSS]) and CAD-RADS score were calculated and compared to clinical indices.

**Results:**

Non-obstructive CAD was found in 109 patients, while 30 patients had a CAD-RADS ≥ 3. Classifying the two groups according to AS, values varied significantly for MFHS (*p* < 0.001), FHRS (*p* < 0.001) and SAFEHEART-RE (*p* = 0.047), while according to SSS only MFHS and FHRS showed significant differences (*p* < 0.001). MFHS, FHRS and SAFEHEART-RE, but not DLCN, showed significant differences between the two CAD-RADS groups (*p* < .001).

MFHS proved to have the best discriminatory power (AUC = 0.819; 0.703–0.937, *p* < 0.001) at ROC analysis, followed by FHRS (AUC = 0.795; 0.715–0.875, *p* < .0001) and SAFEHEART-RE (AUC = .725; .61–.843, *p* < .001).

**Conclusions:**

Greater values of MFHS, FHRS and SAFEHEART-RE are associated to higher risk of obstructive CAD and might help to select asymptomatic patients that should be referred to CCTA for secondary prevention.

**Supplementary Information:**

The online version contains supplementary material available at 10.1007/s11547-023-01610-z.

## Introduction

Familial hypercholesterolemia (FH) is the most common genetic disorder of lipid metabolism characterized by elevated plasma concentrations of low-density lipoprotein cholesterol (LDL-C) [1]. It is caused by mutations in the genes (*LDLR*, *APOB* and *PCSK9*) controlling the receptor-mediated removal of LDL from plasma [1]. FH is inherited as an autosomal co-dominant trait, and most of the patients (1 over 250–300 in the general population) are heterozygotes (HeFH), with only 1 mutated allele [1]. Patients carrying 2 mutated alleles are classified as homozygotes (HoFH) and are much less frequent in the general population (estimated prevalence of 1:160,000 and 1: 360,000) [2].

Individuals with FH are exposed to elevated LDL-C levels since birth so that they should be considered at increased risk of premature atherosclerotic cardiovascular disease (ASCVD). Nevertheless, a considerable number of these vulnerable subjects do not develop cardiovascular events, regardless of the elevated low LDL-C levels [3], while others do so despite intensive lipid-lowering therapy with statins and other medications [4], suggesting that the actual risk is heterogeneous. However, the actual stratification of cardiovascular risk in asymptomatic FH individuals is a challenging task in the clinical setting and cannot be satisfactorily carried out by using the existing CVD risk assessment tools (European SCORE, US Framingham Risk Score [5, 6]).

Three recently introduced clinical scores (Montreal-FH-score [MFHS] [7], SAFEHEART risk equation [SAFEHEART-RE] [8] and FH risk score [FHRS] [9] have been proposed as prognostic tools for ASCVD events in FH, even though both still require validation in different populations [10]. In addition, a crucial aspect that needs to be clarified is the ability of these clinical scores to predict the presence of an increased atherosclerotic burden in asymptomatic FH patients. Furthermore, it has been suggested that the clinical diagnosis of FH should be based on the Dutch Lipid Network (DLCN) score [11]. This score contains several variables (e.g., family history of coronary events and levels of LDL-C) that may also be predictive of atherosclerosis's presence and severity. However, the value of the DLCN score in predicting CAD has never been evaluated.

The demonstration that one or all of these scores can predict in a satisfactory way the early presence of vascular damage in patients with FH would represent their definitive validation as clinical tools to guide therapeutic management of these patents.

Evidence increasingly supports the clinical utility of coronary computed tomography angiography (CCTA) across various stages of CAD, from detecting early subclinical disease to assessing more advanced vascular damage. Additionally, CCTA can be used to noninvasively quantify plaque burden and identify high-risk plaque, aiding in diagnosis, prognosis and treatment [12]. The utility of CCTA has also been demonstrated in FH to accurately estimate the presence and distribution of coronary artery damage [13–15] and to ameliorate long-term prediction of ASCVD events in asymptomatic subjects [16].

Therefore, the aim of our study is to use the CCTA to investigate the actual performance of those clinical scores in predicting CAD extent and severity, and to possibly identify score thresholds that may stratify patients for prompt referral to CCTA.

## Methods

### Study design and population

From October 2013 through May 2019, 139 consecutive patients with clinical diagnosis of FH were prospectively enrolled to perform CCTA in our hospital. They were selected among a population of patients followed at the Lipid Clinic of the Department of Internal Medicine, Sapienza University of Rome, and enrolled into the LIPIGEN-FH Registry.

The LIPIGEN-FH registry is an observational, multicenter, prospective study aimed at identifying and characterizing FH in Italy [17].

In brief, patients were invited to enroll into the registry if they had a clinical diagnosis of “possible,” “probable” or “definite” FH, according to the Dutch Lipid Clinic Network (DLCN) criteria score [18]. Patients were excluded if they were unwilling or unable to sign the informed consent form or had a secondary cause of hypercholesterolemia (e.g., untreated hypothyroidism, nephrotic syndrome or cholestatic liver diseases). All patients with homozygous FH mutations have been excluded from the present study. Patients participating into the study did not undergo any procedure other than normal clinical practice. The study was approved by the Clinical Research Ethics Board of Sapienza University of Rome (approval code #2469), and patients provided written consent.

After enrollment, all FH patients underwent clinical examination and blood drawing for genetic analysis. Blood samples were collected early in the morning after an overnight fast in EDTA-containing tubes. Plasma concentrations of lipoprotein and blood glucose were determined as described previously [19], as well as genetic analysis.

All patients included in the Registry underwent molecular characterization, as previously reported [19]. In brief, genomic DNA was extracted from circulating leukocytes and major candidate genes for FH (*LDLR*, *APOB*, *PCSK9* and *LDLRAP1*) were sequenced by Next Generation Sequencing using MiSeq (Illumina) equipment. Patients where deleterious mutation in FH major candidate gene were definitively identified were classified as monogenic FH. Conversely, those patients in whom monogenic mutations could not be documented were screened by using a weighted polygenic LDL-rising risk score to diagnose the presence of polygenic hypercholesterolemia, as reported elsewhere [19, 20].

Finally, all patients that were (a) asymptomatic for CAD, (b) had nor a past medical history of CAD neither (c) any possible contraindication for CCTA (renal insufficiency, known contrast medium allergy, atrial fibrillation), and (d) were older than 18 years of age were also invited to receive CCTA to evaluate the coronary atherosclerosis burden.

For each patient, DLCN score, MFHS, SAFEHEART-RE (5- and 10-years) and FHRS were calculated [7–9, 11]. Because lipoprotein (a) [Lp(a)] dosage was not available, SAFEHEART-RE and FHRS were re-calculated by considering its value null in all patients.

### Scan protocol and CT analysis

All the exams were performed using a first generation dual-source CT scanner (SOMATOM Definition, Siemens Healthcare, Erlangen, Germany). Before undergoing CCTA, all patients with suboptimal heart rate received beta-blocker therapy (oral metoprolol a few days before the examination and/or intravenous esmolol just before scan) with a target heart rate < 70 bpm.

In patients with no contraindications, nitrates (isosorbide dinitrate, 5 mg) were administered sublingually 5–10 min before the exam to achieve optimal coronary vasodilation.

All exams were ECG-gated, either retrospectively or prospectively, the decision being taken singularly for each patient depending on patient’s heart rate.

A preliminary prospectively ECG-gated non-contrast scan was performed for the assessment of coronary artery calcium (CAC). The entire heart was covered by the non-contrast CT scan, which started at the aortic root level above the coronary ostia; the ECG-gated slices were obtained during single breath hold with the following scanning parameters: 4 × 2.5 mm collimation; slice thickness 3 mm; slice increment 1.5 mm; 120 kV, 100 mAs, 0.33 s gantry rotation time and prospectively ECG triggering at 70% of R-R interval.

A bolus of 80–90 ml of high-concentrated iodinated contrast agent (Iomeprol 400 mgI/100 mL, Iomeron 400, Bracco, Milan, Italy) was administered at high flow rate (5.0 ml/s), followed by 50 mL of saline flush.

All images were analyzed by using a dedicated workstation (Vitrea, Vital Images, Minnetonka, MN).

The Agatston score (AS), Volume and Mass of CAC were calculated for each study.

Segmentation of the coronary tree was performed according to the 17-segments American Heart Association classification, and all segments with a diameter of 2 mm or more were included in the analysis. Degree of luminal stenosis was classified for each coronary segment as normal (no plaque), minimal (< 25% stenosis), mild (25–49% stenosis), moderate (50–69% stenosis), severe (70–99% stenosis), occluded (complete occlusion), according to the Society of Cardiovascular Computed Tomography (SSCT) recommended guidelines [21]. All images were analyzed independently by 2 readers, and when disagree occurs, the exam was re-evaluated in a joined session to achieve a consensus. To avoid any possible bias, radiologists were blinded for genotype and clinical scores.

A qualitative assessment of plaque composition was also made, and Plaque Composition Sum (PCS) was calculated for each patient, by summing a value associated with plaque composition for each one of the patient’s plaques (1: calcified, 2: mixed, 3: soft).

**Fig. 1.**
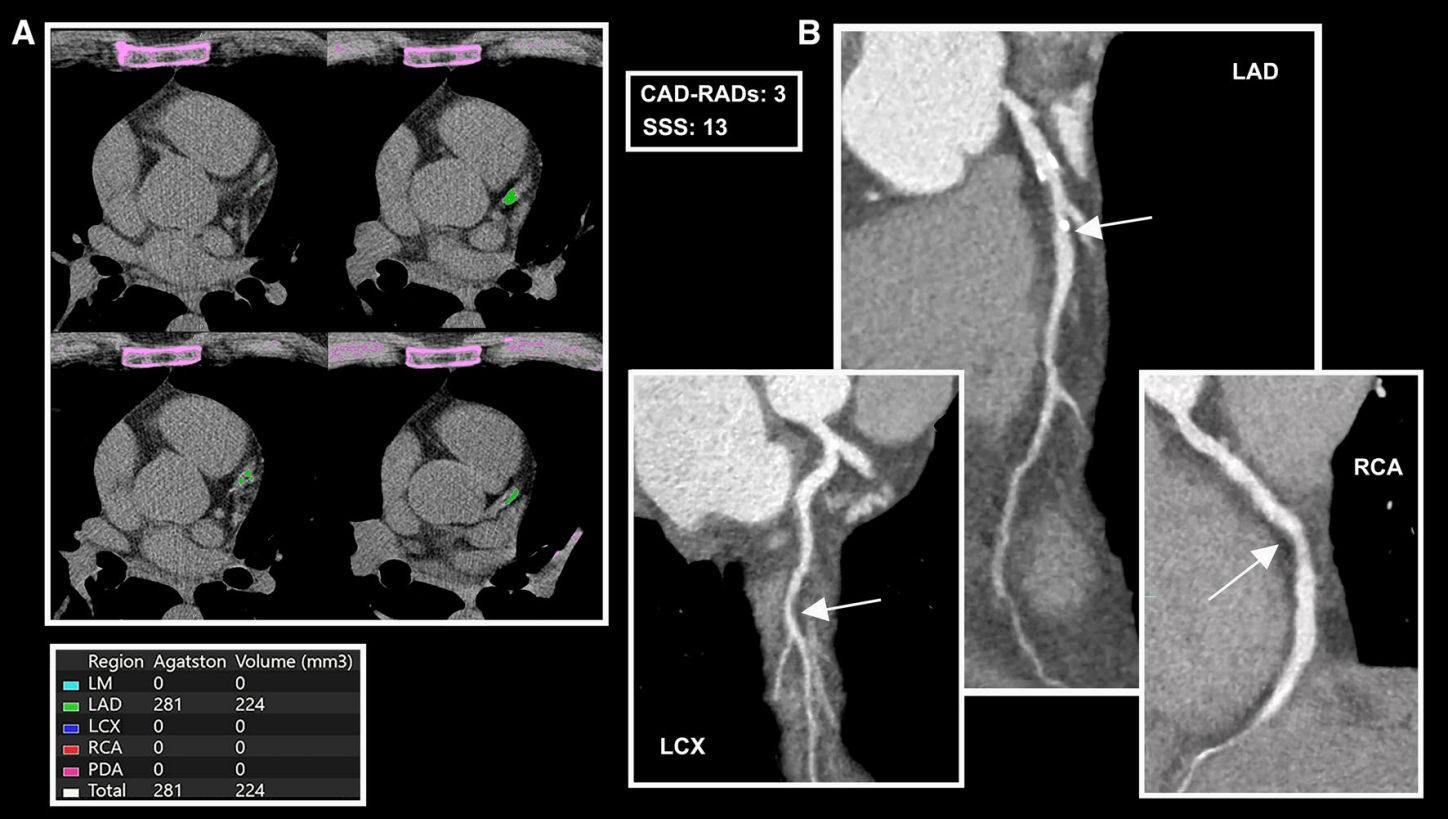
49-year-old male with obstructive CAD. CAC scoring evaluation (**A**) show Agatston score = 282 (class 2). CPR (**B**) show coronary atherosclerotic involvement (SSS = 13): one mixed plaque causing moderate stenosis in the middle segment of LAD (white arrow), one soft plaque causing moderate stenosis in LCX (white arrow) and one soft plaque causing mild stenosis in RCA (CAD-RADS = 3) Clinical scores were: DLCN = 13, MFHS = 26, SAFEHEART-RE 5y = 1.23, SAFEHEART-RE 10y = 2.6. *CAC: coronary artery calcium; CPR: curved planar reconstruction; DLCN: Dutch Lipid Clinic Network score; LAD: left descending artery; LCX: left circumflex artery; MFHS: Montreal-FH-score PCS: plaque composition sum; RCA: right coronary artery; SAFEHEART-RE: SAFEHEART Risk Equation; SSS: segment stenosis score*

Atherosclerotic burden was calculated at CCTA for each patient using segment stenosis score (SSS) and Coronary Artery Disease Reporting and Data System (CAD–RADS) score [22], according to the validated approach (Fig. 1).

The segment stenosis score (SSS) was calculated summing the scores given to each segment depending on the severity of the local stenosis (0: normal, 1: minimal stenosis, 2: mild stenosis, 3: moderate stenosis, 4: severe stenosis or occluded vessel).

Based on CAD-RADS score, FH patients were classified in two groups: CAD-RADS ≤ 2 and CAD-RADS ≥ 3, as a CAD-RADS value higher than 3 (at least one stenosis with moderate degree) is consistent with obstructive disease and clinically relevant for treatment.

### Statistical analysis

The data, unless otherwise stated, were given as mean with standard deviation (± SD) or medians with interquartile ranges (25-th and 75-th percentile) for continuous variables and as simple frequencies, proportions, and percentages for categorical variables. Variables with significant differences were tested by a binary logistic analysis together with the score and presented with a 95% Confidence Interval (CI). Continuous variables were compared by Student t test, or ANOVA, Mann–Whitney and Kruskal–Wallis tests if normally or not-normally distributed, respectively; categorical variables were compared by Fisher’s exact tests or Chi-square statistics.

To assess the discriminatory power of the clinical score for predicting presence of clinically relevant CAD (CAD-RADS ≥ 3), we performed and compared the receiver operating characteristic (ROC) curve analysis of DLCN, MFHS, FHRS and SAFEHEART-RE 5- and 10-years scores. Area under the curve (AUC) range from 0.5 to 1.0; a measure of 0.5 indicates that the discrimination is caused by chance alone, and 1.0 indicates perfect discrimination. P value analyses were two-sided, and a p value of less than 0.05 was considered statistically significant. Youden’s test was applied to identify the optimal cutoff for each clinical score. The Positive Predictive Values (PPV) and the Negative Predictive Values (NPV) were calculated to evaluate the proportions of a true positive and negative results. The logarithm of the AS [LOG(AS + 1)] was used, according to previous studies [23], to test the benefit of CAC evaluation in improving clinical score predictive value. Net reclassification improvement (NRI) was calculated to assess the discrimination improvement by comparing the AUCs in models with MFHS, FH risk score and SAFEHEART-RE 5y alone and combined with log(AS + 1). All statistical analyses were performed with Statistical Package for Social Science (SPSS) version 25, which is a graphical user interface for MATLAB (ver. 2021).

## Results

### Clinical characteristics and CCTA features

Clinical characteristics, laboratory data and cardiac CT findings of enrolled FH patients are summarized in Table 1. As a whole, they appeared to be young, mainly male and thin, and 85% reported previous lipid-lowering treatments; the most common risk factors were arterial hypertension and smoking. Moreover, 72% presented confirmed monogenic FH, due to mutations in the LDLR gene, and 28% were classified as affected by polygenic hypercholesterolemia.

**Table 1:** Patients’ characteristics and CCTA features

Clinical and laboratory parameters	Values
Age, mean (SD)	48 (12.9)
Gender male, No. (%)	82 (59)
BMI, mean (SD)	25.6 (3.5)
Time interval from diagnosis in month, median (IQR)	25 (13.3–34)
LLT, No. (%)	118 (85)
LLT duration in months, median (IQR)	48 (22.8–112.3)
*Genetic, No. (%)*	
Monogenic	100 (72%)
Polygenic	39 (28%)
*CV Comorbidities, No. (%)*	
Hypertension	29 (21)
Diabetes	5 (4)
Smoking	72 (52)
*Laboratory findings, median (IQR)*	
Glycemia, mg/dl	90 (83–97)
LDL cholesterol	135.2 (106.3–181.9)
HDL cholesterol	55 (46–64)
Total triglycerides	97 (72.5–126.5)
*Clinical scores, mean (SD)*	
DLCN	12.9 (5.9)
Montreal risk score	22.7 (7.6)
SAFEHEART-RE 5y	0.9 (0.9)
SAFEHEART-RE 10y	1.9 (1.9)
FH risk score	24.6 (9.5)
*CCTA features*	
Agatston score, mean (SD)	151 (342)
*Agatston class, No (%)*	
0 = 0	71 (51)
1 = 1–100	34 (24)
2 = 101–300	19 (14)
3 = > 300	15 (11)
SSS, mean (SD)	4 (6.2)
PCS, mean (SD)	3.4 (4.6)
*CAD-RADS, No (%)*	
0	63 (45.3)
1	19 (13.7)
2	27 (19.4)
3	18 (12.9)
4	10 (7.2)
5	2 (1.4)

*BMI* body mass index*, CAC *coronary artery calcium*, CV* cardiovascular,* DLCN* Dutch Lipid Clinic Network score,* FH* familial hypercholesterolemia,* LLT* Lipid-lowering therapy,* MFHS *Montreal-FH-score,* PCS* plaque composition sum, *SAFEHEART-RE* SAFEHEART risk equation, *SD*: standard deviation, *SSS* segment stenosis score

Mean DLCN was 12.9, MFHS was 22.7, FHRS was 24.6, and SAFEHEART-RE was 0.9 at 5 years and 1.9 at 10 years.

**Fig. 2:**
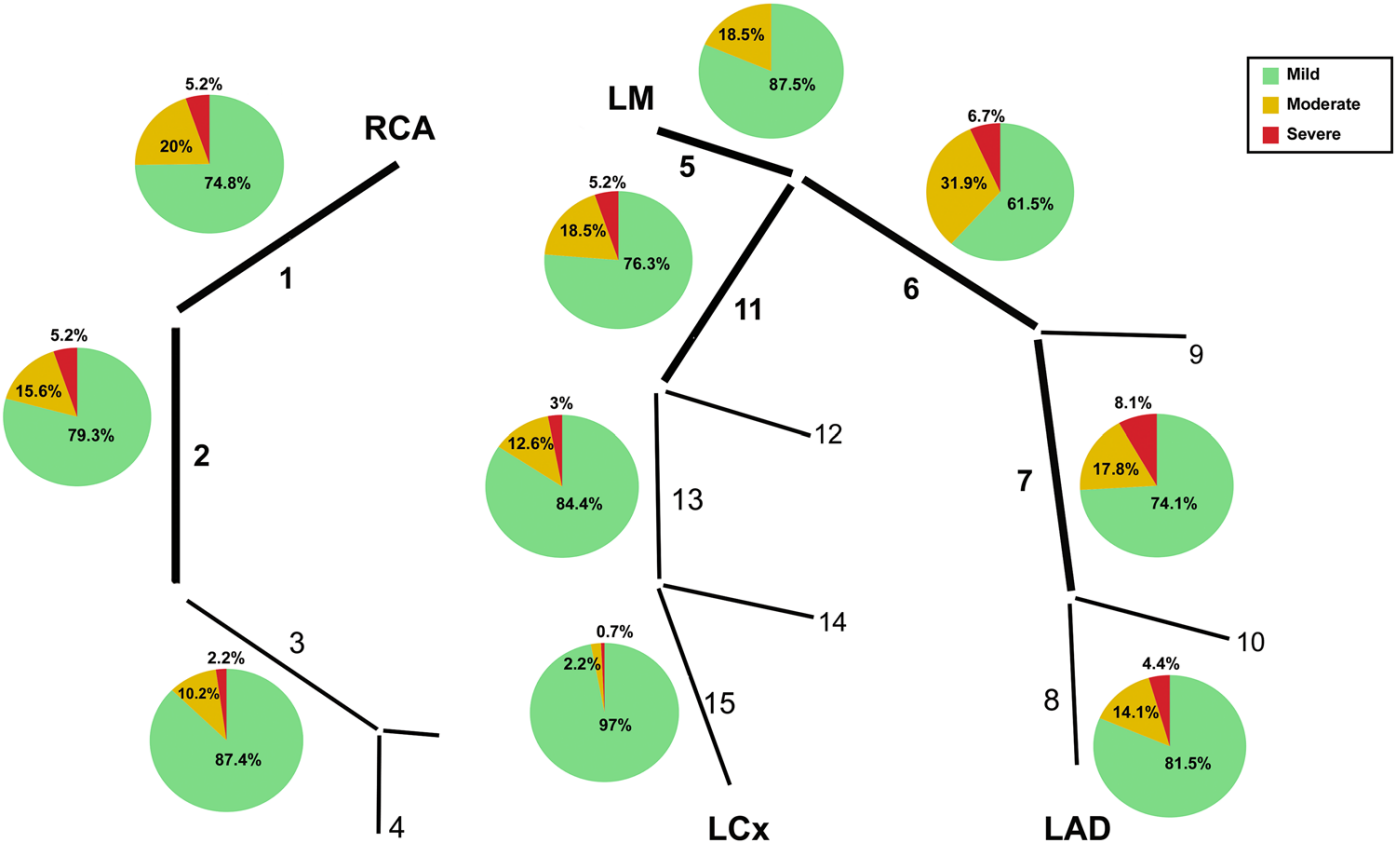
Presence and severity of coronary plaques as distributed in the proximal, mid and distal segments of the coronary tree, according to AHA segmentation scheme. *LAD: left anterior descending coronary artery; LCX: left circumflex coronary artery; LM: left main; RCA: right coronary artery*

CAC was higher than zero in about half of patients (49.6%), and average AS was 151.01 (± 342.04) units. CCTA findings showed 54.7% of the patients with at least one plaque detected, and mean SSS was 4 (± 6.23). Plaque composition analysis showed that out of the 318 plaques detected at CCTA, 171 (54%) were calcified, 102 (32%) were mixed, and 45 were soft (14%), with a mean PCS of 3.4 (± 4.6). The distribution of plaques along the coronary tree of our patients is depicted in Fig. 2: The proximal left anterior descending artery was the most affected segment, and the proximal segments were globally more involved than the mid and distal ones.

Non-obstructive CAD was found in 109 patients, classified as CAD-RADS 0–2, while 30 patients had a CAD-RADS ≥ 3. The youngest patient showing an obstructive disease (CAD-RADS 3) was a 33-year-old patient.

The clinical and CCTA characteristics of patients are summarized according to CAD-RADS in Table 2. FH patients with CCTA findings of obstructive disease (CAD-RADS ≥ 3) were older (*p* < 0.001), had higher BMI (*p* < 0.042) and were more likely to have diabetes (*p* = 0.001), hypertension (*p* = 0.017), higher values of triglycerides (*p* = 0.041) and longer time interval from diagnosis (*p* = 0.021). Multivariate predictors of obstructive CAD were age (*p* < 0.001), sex (*p* = 0.022), diabetes (*p* = 0.006) and the time interval from diagnosis (*p* = 0.014); neither lipid-lowering therapy, nor cholesterol (both HDL and LDL) nor total triglycerides resulted as significant predictor of elevated CAD-RADS at the multivariate analysis. Soft plaques were significantly more represented in the group with obstructive disease (PCS 1.66 ± 2.79 vs 9.63 ± 4.52, *p* < 0.001).

**Table 2:** Clinical parameters in CAD-RADS groups

	CAD-RADS ≤ 2	CAD-RADS ≥ 3	*P* value
*Clinical and laboratory parameters*			
Age, mean (SD)	46.5 (12.5)	57.3 (11.14)	** < *0.001***
Gender male, No. (%)	60 (55)	22 (73.3)	0.072
BMI, mean (SD)	25.4 (3.6)	26.6 (3.2)	***0.042***
Monogenic FH, No. (%)	76 (69.7)	24 (80)	0.271
*CV Comorbidities, No. (%)*			
Hypertension	18 (16.7)	11 (36.7)	***0.017***
Diabetes	1 (0.9)	4 (13.3)	***0.001***
Smoking	54 (50)	18 (60)	0.336
*Laboratory findings, median (IQR)*			
Glycemia, mg/dl	89 (83–95)	93 (84.5—103)	***0.039***
LDL cholesterol	135.8 (106.4–180.8)	133.9 (105.9–187)	0.471
HDL cholesterol	56 (48–64)	51 (46–59)	0.156
Total triglycerides	95 (70–117)	104 (81–159)	***0.041***
Time interval from diagnosis in month (mean ± SD)	24 (13–32)	34 (18.5–41.5)	***0.021***
LLT, No. (%)	91 (83.5)	27 (90)	0.381
LLT duration in months, mean (SD)	72.3 (71.8)	112 (112.5)	***0.036***
*Clinical scores, mean (SD)*			
DLCN	12.8 (6.2)	13.2 (4.8)	0.762
Montreal risk score	20.9 (7.1)	29 (5.8)	** < *0.001***
SAFEHEART-RE 5y	0.78 (0.9)	1.39 (0.9)	** < *0.001***
SAFEHEART-RE 10y	1.64 (1.8)	2.92 (1.9)	** < *0.001***
FH risk score	22.58 (9.1)	31.9 (7.1)	** < *0.001***
*CCTA features*			
CAC score > 0, No. (%)	39 (35.8)	29 (96.7)	** < *0.001***
Agatston score, mean (SD)	37.7 (82.9)	572.9 (536.4)	** < *0.001***
SSS, mean (SD)	1.4 (2.3)	13.1 (7.2)	** < *0.001***
PCS, mean (SD)	1.6 (2.8)	9.6 (4.5)	** < *0.001***

*BMI* body mass index, *CAC* coronary artery calcium, *CV* cardiovascular, *DLCN* Dutch Lipid Clinic Network score, *FH* familial hypercholesterolemia, *LLT* Lipid-lowering therapy, *MFHS* Montreal-FH-score, *PCS* plaque composition sum, *SAFEHEART-RE* SAFEHEART Risk Equation, *SD* standard deviation, *SSS* segment stenosis score; *p* values were considered significant when < 0.05

Moreover, patients showed significant differences in AS when categorized and compared by the presence of hypertension (118.49 ± 322.17 vs 281.83 ± 393.87; *P* = 0.024), and diabetes mellitus type 2 (132.35 ± 328.92 vs 670.07 ± 328.34; *p* < 0.001); no differences were found when compared by lipid-lowering therapy (26.05 ± 70.95 vs 173.63 ± 366.21; *p* = 0.069) and smoking (174.96 ± 412.83 vs 131.22 ± 264.99; *p* = 0.460). All the results are summarized in Supplementary Material.

### Performance of clinical scores in predicting CAD

**Fig. 3:**
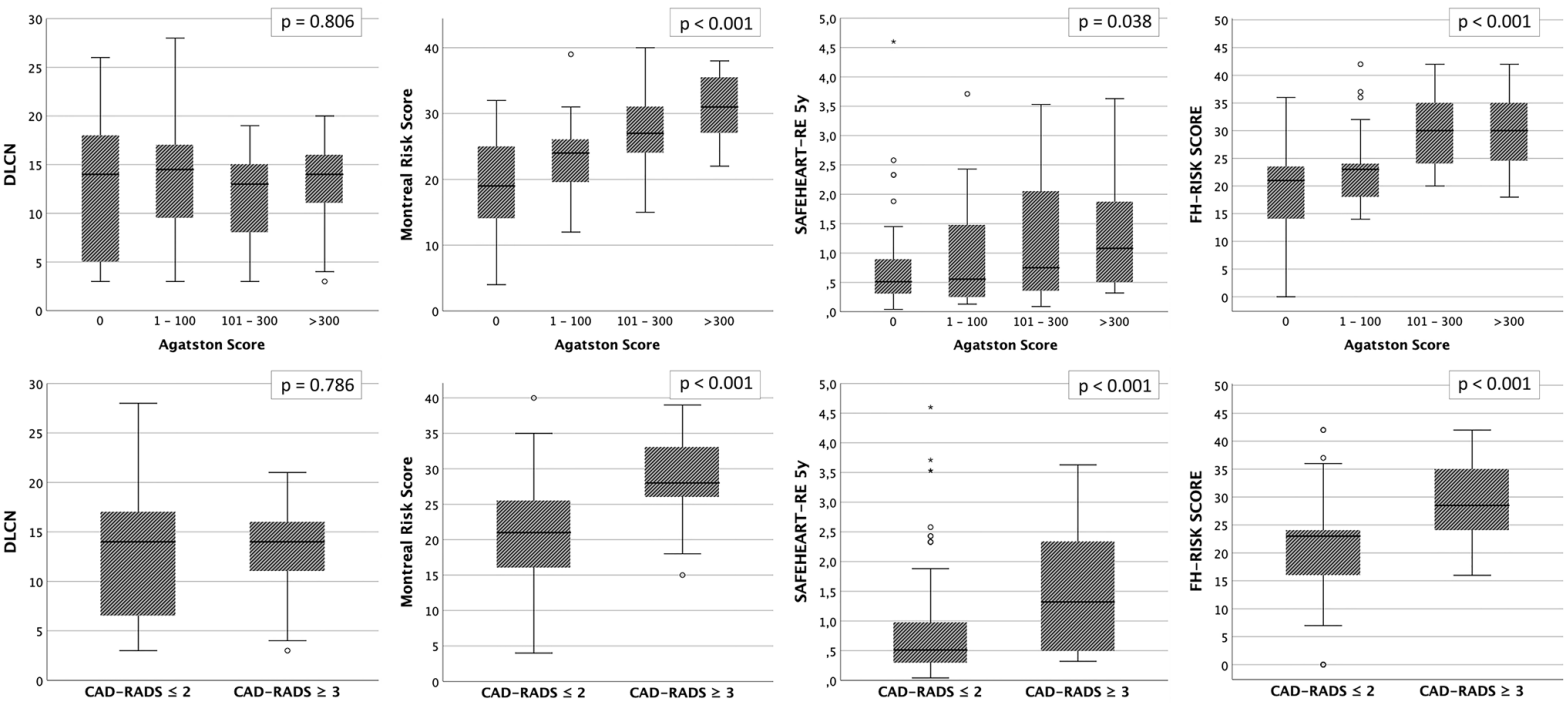
Box plot graphs of DLCN, Montreal Risk Score, FHRS and SAFEHEART-RE comparing population divided by AS and CAD-RADS Montreal Risk Score, FHRS and SAFEHEART-RE at 5 years showed significant difference in AS groups (*p* < 0.001, *p* < 0.001 and *p* = 0.038, respectively) and CAD-RADS groups (*p* < 0.001 for all the scores). DLCN showed no significant difference in any population subdivision (*p* = 0.786 in CAD-RADS groups and *p* = 0.806 in AS groups). In all the box plots, the top of the box represent the third quartile and the bottom the first quartile. The horizontal line represents the median for entire cohort. The whiskers go from each quartile to the minimum or maximum. *AS: Agatston score; DLCN: Dutch Lipid Clinic Network Criteria; SAFEHEART-RE: SAFEHEART Risk Equation; SSS: segment stenosis score*

Classifying the study population based on CAD severity according to AS (0, 1–100, 101–300, > 300), we observed that values varied significantly for MFHS (*p* < 0.001), FHRS (*p* < 0.001) and SAFEHEART-RE (*p* = 0.047), whereas DLCN did not show statistically significant differences (*p* = 0.786, Fig. 3). However, dividing the population by SSS values, only MFHS and FHRS showed significant differences (*p* < 0.001).

MFHS, SAFEHEART-RE and FHRS were significantly different between the two CAD-RADS groups (*p*: < 0.001 for all), unlike the DLCN (p: 0.806).

**Fig. 4:**
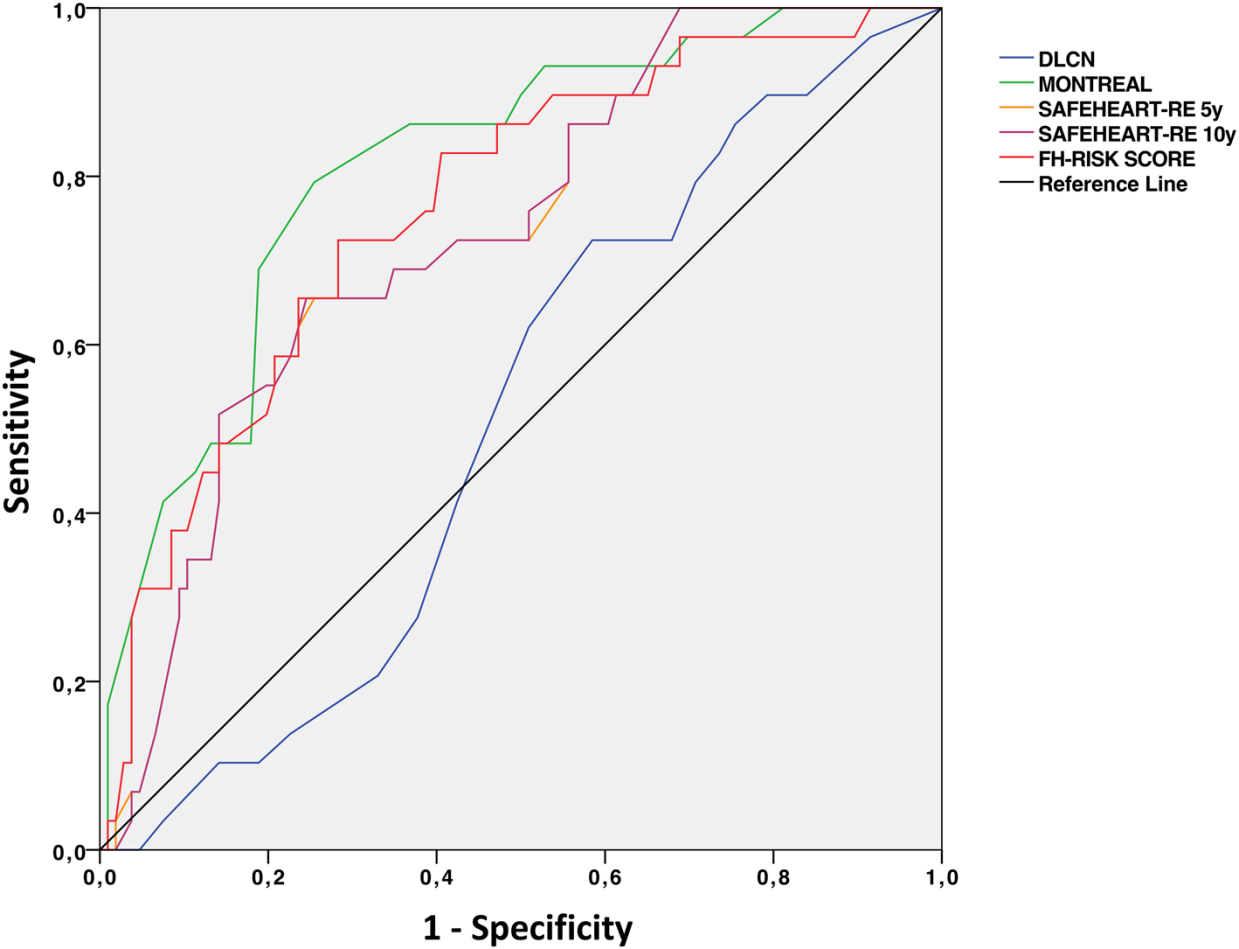
Receiving Curve Analysis of Clinical Scores in predicting obstructive CAD ROC curve illustrates the diagnostic performance DLCN (blue), Montreal score (yellow) and SAFEHEART-RE (orange at 5 years and green at 10 years) to predict CAD-RADS ≧ 3 at CCTA. Montreal score shows the best performance (AUC = 0.819; 95% confidence interval: 0.703–0.937, *p* < 0.001), followed by FHRS (AUC = 0.795; 0.715–0.875, *p* < 0.001) and SAFEHEART-RE (AUC = 0.725; 95% confidence interval: 0.61–0.843, *p* < 0.001), whereas DLCN showed very poor performance (AUC = 0.517; 95% confidence interval: 0.398–0.636, *p* = 0.690) *CAD: coronary artery disease; CCTA: coronary computed tomography angiography; DLCN: Dutch Lipid Clinic Network Criteria*

ROC analysis (Fig. 4) showed that DLCN failed to discriminate between the groups (AUC = 0.517), while MFHS proved to have a good discriminatory power excellent (AUC = 0.819; 95% confidence interval: 0.703–0.937, *p* < 0.001), followed by FHRS (AUC = 0.795; 0.715–0.875, *p* < 0.001) and SAFEHEART-RE at 5 y (AUC = 0.725; 95% confidence interval: 0.61–0.843, p < 0.001).

The best cutoffs for the distinction between non-obstructive Vs obstructive CAD groups were 25.5 for MFHS (sensitivity or Se: 79.3%, specificity or Sp: 74.5%), 25.5 for FHRS (Se: 86.7%, Sp: 64.5%), 0.93 for SAFEHEART-RE at 5 years (Se: 65.5%, Sp: 74.5%) and 2.06 at 10 years (Se: 65.5%, Sp: 75.5%).

The LOG(AS + 1) was calculated for each patient and added to MFHS, FH risk score and SAFEHEART-RE 5y, obtaining “improved risk scores.” The resulting improvement of obstructive CAD risk prediction was calculated as difference in AUCs and reported in *Supplementary Materials*. In particular, the clinical score with the greatest improvement after the correction for CAC score was the SAFEHEART-RE at 5y (AUC: 0,725 Vs. 0,905; *p* < 0.001).

## Discussion

In line with previous investigations [15, 24, 25], asymptomatic FH patients included in the present a cohort showed high prevalence and wide extension of CAD, reaffirming the notion that FH must be considered a high-risk group for premature atherosclerotic vascular damage.

Moreover, in our study, we investigated the performance of the most accepted ASCVD risk stratification scores, the MFHS, the FHRS and SAFEHEART-RE, in predicting CAD extent and severity. We demonstrated that while the traditional clinical disease staging with DLCN score does not correlate with the severity of CAD detected by CCTA, higher values of MFHS, FHRS and SAFEHEART-RE proved to be associated with increased prevalence of obstructive CAD.

More specifically, higher values of MFHS, FHRS and SAFEHEART-RE were associated with increased burden of CAC with greater statistical significance with the MFHS and FHRS (*p* < 0.001 for both), than SAFEHEART-RE (*p* = 0.038), whereas only MFHS and FHRS were able to predict subclinical CAD, indicated by higher SSS (*p* < 0.001).

These results are only partially in line with previous findings of the SAFEHEART investigators [15] who demonstrated that both the CAC score and SSS were independently correlated with the risk calculated by applying their score.

In our study, the combination of CAC score with clinical scores was able to significantly improve the predicting model and allowed the correct upward reclassification of 87% of patients with obstructive CAD and downward reclassification of 30% in patients with non-obstructive CAD, for all the scores (see ﻿Supplementary Material online only﻿).

Although the AS and SSS are validated as prognostic indices due to their relationship with mortality and coronary events, they show several limitations in guiding clinical management.

Indeed, AS doesn’t take into account the presence of soft plaques, and even if several studies [24, 25] suggested that it may be useful in ASCVD risk stratification, it cannot exclude reliably the presence of obstructive CAD. In this regard, in our population soft plaques were significantly more represented in the group with obstructive CAD and were the cause of complete vessel obstruction in two patients.

On the other hand, SSS can correspond to extremely different plaque distribution patterns with different clinical therapeutic management (e.g., SSS = 5 could indicate complete occlusion of the proximal left anterior descending artery requiring urgent revascularization and 5 mild, calcified, peripheral stenosis only requiring risk factor control) [26].

Therefore, we decided to include in our study the CAD-RADS, a score designed with the aim of helping clinical management [12] that relies on the most severe plaque to promptly refer patients to ICA. We divided our population according to CAD-RADS score into two groups: 109 patients with non-obstructive CAD (CAD-RADS < 3) and 30 patients with obstructive CAD (CAD-RADS ≥ 3).

In our results, MFHS, FH-score and SAFEHEART-RE, unlike DLNC, showed significant differences between the two CAD-RADS groups (*p* < 0.001).

Although a reduction in the relative risk of CAD was observed in previous studies due to statin therapy [4], the multivariate analysis that we performed in our population did not reveal a significant prediction of obstructive CAD as regards lipid-lowering therapy. Instead, clinical multivariate predictors of elevated CAD-RADS were represented by age, sex, diabetes and the time interval from the diagnosis.

MFHS showed the greatest AUC (i.e., the highest predictive value) of 0.819, whereas FHRS and SAFEHEART-RE at 5-years had AUC of 0.795 and 0.725, respectively. As described above, the predictive performance of all clinical scores has been improved by the integration of the CAC score within the formula, with the most significant benefit for SAFEHEART-RE at 5-years (AUC from 0.725 to 0.905).

The best cutoffs for the distinction between the non-obstructive and the obstructive CAD group were 25.5 for MFHS and FHRS, and 0.93 for SAFEHEART-RE at 5 years.

Our results proved that MFHS, FHRS and SAFEHEART-RE might have a role in selecting patients at higher risk of increased atherosclerosis burden, who would benefit from early assessment of coronary arteries by CCTA.

The DLCN is a score created to classify patients based on the likelihood of a diagnosis of FH, which does not take into account clinical variables critical to cardiovascular risk assessment, e.g., gender or hypertension, so it is not surprising that it could not effectively identify patients with obstructive CAD.

Regarding the different performances of the specific ASCVD scores, some considerations are needed. First of all, leaps to the eye that both FHRS and SAFEHEART-RE, which performed slightly less well than the MFHS, include LDL-C value, which was not shown to be significant in detecting patients with obstructive CAD on multivariate analysis in our population.

Moreover, it can be speculated that the lack of LP(a) values, a major limitation of the study, negatively affected the performance of both FHRS and SAFEHEART-RE compared to MFHS. CCTA can play a key role in the management of these patients, allowing both risk stratification of cardiovascular events [27–29] and early detection of obstructive CAD requiring prompt therapeutic intervention. The latest update of European Society Cardiology (ESC) guidelines on cardiovascular disease prevention in clinical practice [30] acknowledges the role of CCTA in identifying coronary stenosis and predicting cardiac events but does not provide guidance on its use in the general population nor the FH population.

Although the International Atherosclerosis Society has previously recommended CCTA in severe FH, a diagnostic pathway has not yet been defined [3].

Algorithms combining clinical scores and CCTA could be formulated to warrant a tailoring of patient management.

In this regard, we believe that the cutoff values of clinical scores for addressing to CCTA should privilege high values of sensitivity, given the importance of early and prompt detection of obstructive CAD in this population.

Therefore, in our opinion, the cutoff values of 21.5 (Se: 89.7% and Sp: 50%) for MFHS, 0.32 for SAFEHEART-RE at 5 years (Se: 89.7% and Sp: 38.7%) and 22.5 for FHRS (Se: 89.7% and Sp: 54.7%) would be more appropriate.

To the best of our knowledge, this is the first study to evaluate the performance of ASCVD risk clinical scores in predicting coronary atherosclerosis burden detected by CCTA in a 100% molecularly defined FH cohort.

It is a common opinion that current ASCVD risk stratification strategies in FH are still unsatisfactory [31] and should be implemented by combining clinical-laboratory data with imaging features for better phenotyping.

### Limitations

We recognize that several limitations affect our study. The small size and heterogeneity of our population. The low number of patients with obstructive CAD may represent a limitation, even though its prevalence (21.6%) was consistent with those reported in previous studies on asymptomatic subjects, ranging from 17% in the general population [32] to 23% in diabetic patients [33]. Nevertheless, validation of these data on larger cohorts is mandatory.

The lack of Lp(a) values may adversely affect the performance of the SAFEHEART-RE and FHRS. We recognize this as a major limitation of our study; however, we believe that this cutoff may still be of added value in clinical practice, where Lp(a) dosing is not always available.

The population was heterogenous regarding the lipid-lowering therapy in terms of drugs and duration, which is not included in clinical scores calculation.

The lack of up-to-date CT technology to ensure better stratification of patients with moderate stenosis (e.g., CT myocardial perfusion, fractional flow reserve-CT [FFR-CT]).

Finally, the lack of clinical follow-up dictates further studies with larger populations to assess the actual impact of CCTA in the management of CAD in FH.

## Conclusions

CCTA enables early detection of the presence, extent and severity of CAD. MFHS, FH-score and SAFEHEART-RE proved to be able to predict obstructive CAD and might help to select patients that should be referred to CCTA. Further studies in larger populations are needed to confirm these findings and to improve risk stratification systems and diagnostic pathways.

## Supplementary Information

Below is the link to the electronic supplementary material.

Supplementary file1 (DOCX 490 kb)
