# Supplementary material for: Effectiveness of clinical scores in predicting coronary artery disease in familial hypercholesterolemia: a coronary computed tomography angiography study
Source: Radiol Med. 2023 Mar 6;128(4):445–55. doi: 10.1007/s11547-023-01610-z (PMC10119045; doi:10.1007/s11547-023-01610-z)
Supplement: Supplementary file 1 — Supplementary file1 (DOCX 490 kb) [file 11547_2023_1610_MOESM1_ESM.docx]

**Supplementary Materials**

**Comparison of CAC scoring by clinical risk factors**

Box-Plots showing CaCs values among patients when categorized by cardiovascular risk factors.


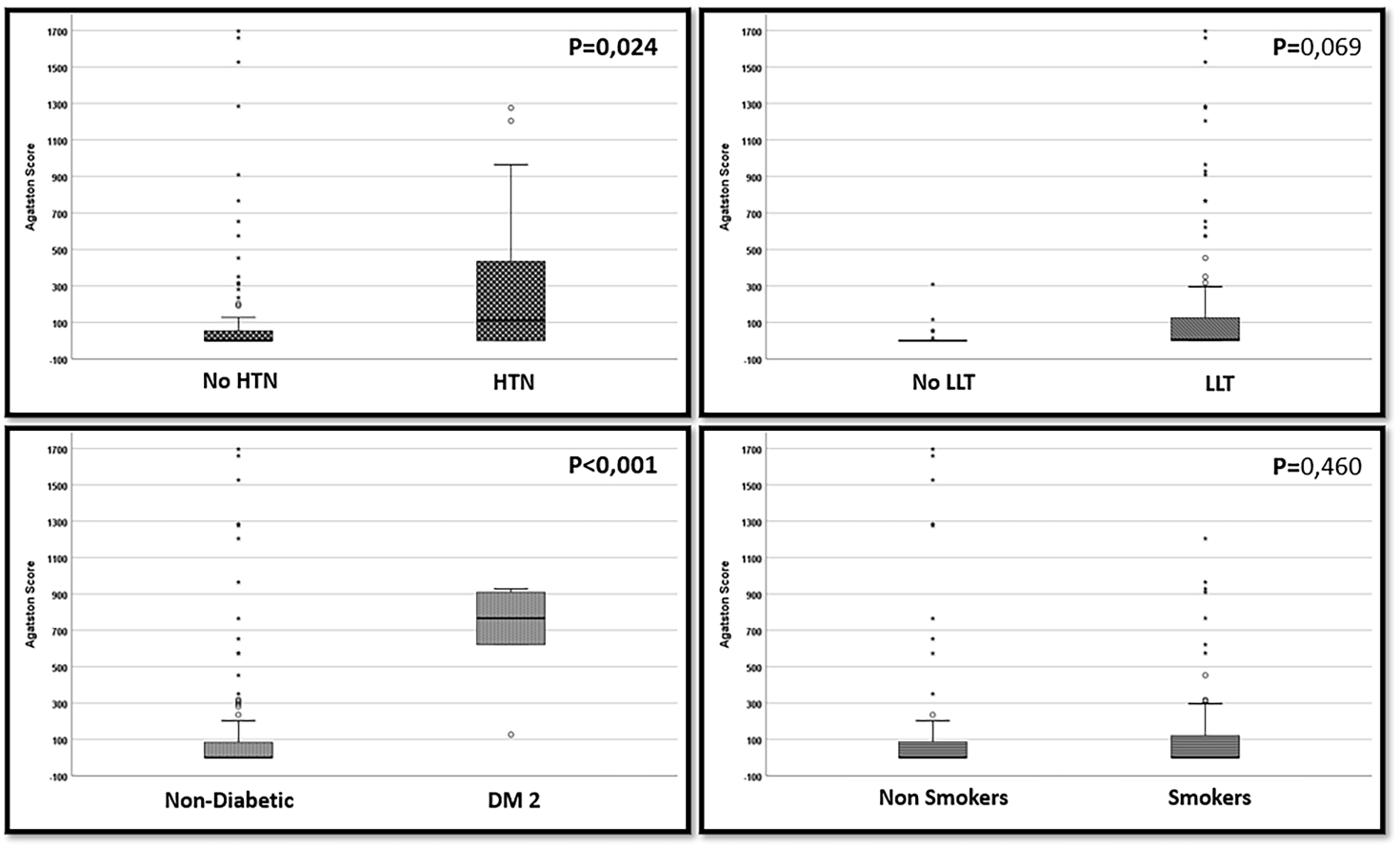


CaCs showed significant differences between patients when categorized for Hypertension (118.49±322.17 vs 281.83±393.87; P=0.024), and Diabetes Mellitus type 2 (132.35±328.92 vs 670.07±328.34; p<0.001); no differences were found when categorized for Lipid-lowering therapy (26.05±70.95 vs 173.63±366.21; p=0.069) and Smoking (174.96±412.83 vs 131.22±264.99; p=0.460)

**CaCs values in FH patients when categorized for Cardiovascular Risk factors**

| Score | Absent (mean±SD) | Present (mean±SD) | P value |
| --- | --- | --- | --- |
| HTN | 118.49±322.17 | 281.83±393.87 | **.024** |
| DM2 | 132.35±328.92 | 670.07±328.34 | **<.001** |
| LLT | 26.05±70.95 | 173.63±366.21 | **.069** |
| Smoking | 174.96±412.83 | 131.22±264.99 | **.460** |

The analysis was performed using one-way ANOVA. Statistically significant values are considered for p<0.05.

The LOG(CaC+1) was calculated for each patient using the Agatston Score and then added to MFHS, FH risk score, and Safeheart-RE 5y, obtaining “improved risk scores”, and allowing for improving the redistribution of patients’ risk. All the results are reported in Table below.

**Performance of Clinical Risk Scores combined with CAC scoring in predicting obstructive CAD**

LOG(AS+1) and Improved Risk Scores values in non-obstructive and obstructive CAD patients

| Score | NOCAD (mean ± SD) | OCAD (mean±SD) | P value |
| --- | --- | --- | --- |
| LOG(AS+1) | 0.57±0.86 | 2.39±0.83 | **<.001** |
| MFHS + Log(AS+1) | 21.46±7.54 | 31.42±6.04 | **<.001** |
| FH risk score + Log(AS+1) | 23.11±9.51 | 34.29±7.27 | **<.001** |
| Safeheart RE 5y + Log(AS+1) | 1.36±1.33 | 3.77±1.25 | **<.001** |

*NOCAD:* non-obstructive CAD; *OCAD:* obstructive CAD.

The discrimination between NOCAD and OCAD patients was improved in all clinical risk scores when CaC score was added.

ROC curves comparing MHFS, FH risk score, SAFEHEART-RE 5y, Log(CAC+1) alone and combined, for the risk prediction of Obstructive CAD

The AUCs with P-value and CI 95%, are reported in the following Table.

Performance of Clinical Risk Scores and Clinical Scores improved with CAC

| Score | AUC | P value | Confidence interval 95% | |
| --- | --- | --- | --- | --- |
|  |  |  | **Lower limit** | **Upper limit** |
| *MFHS* | 0,819 | 0,000 | 0,703 | 0,937 |
| *MFHS + Log(AS+1)* | 0,856 | 0,000 | 0,784 | 0,929 |
| *FH-risk score* | 0,795 | 0,000 | 0,715 | 0,875 |
| *FH-risk score + Log(AS+1)* | 0,833 | 0,000 | 0,762 | 0,904 |
| *SAFEHEART-RE 5 y* | 0,725 | 0,000 | 0,610 | 0,843 |
| *SAFEHEART RE 5 y + Log(AS+1)* | 0,905 | 0,000 | 0,853 | 0,956 |
| *LOG(AS+1)* | 0,915 | 0,000 | 0,848 | 0,981 |

Net Reclassification Improvement (NRI) Table

|  |  | *nup* | *ndown* | *N* |  |  |
| --- | --- | --- | --- | --- | --- | --- |
| ***Events*** | *nup* | *28* | *2* | *30* | ***NRIevents*** | *87%* |
| ***NonEvents*** | *ndown* | *38* | *70* | *108* | ***NRInon-events*** | *30%* |

The integration of CAC scoring allowed a correct upward reclassification of 87% of OCAD patients and a correct downward reclassification of 30% of NOCAD patients for all the scores. The Overall NRI was 1.16.
